# Self-organizing Complex Networks: individual versus global rules

**DOI:** 10.3389/fphys.2017.00478

**Published:** 2017-07-07

**Authors:** Korosh Mahmoodi, Bruce J. West, Paolo Grigolini

**Affiliations:** ^1^Center for Nonlinear Science, University of North TexasDenton, TX, United States; ^2^Army Research OfficeResearch Triangle Park, NC, United States

**Keywords:** network dynamics, self-organized temporal criticality, evolutionary game theory, emergence of cooperation, swarm intelligence

## Abstract

We introduce a form of Self-Organized Criticality (SOC) inspired by the new generation of *evolutionary game theory*, which ranges from physiology to sociology. The single individuals are the nodes of a composite network, equivalent to two interacting subnetworks, one leading to strategy choices made by the individuals under the influence of the choices of their nearest neighbors and the other measuring the Prisoner's Dilemma Game payoffs of these choices. The interaction between the two networks is established by making the imitation strength *K* increase or decrease according to whether the last two payoffs increase or decrease upon increasing or decreasing *K*. Although each of these imitation strengths is selected selfishly, and independently of the others as well, the social system spontaneously evolves toward the state of cooperation. Criticality is signaled by temporal complexity, namely the occurrence of non-Poisson renewal events, the time intervals between two consecutive crucial events being given by an inverse power law index μ = 1.3 rather than by avalanches with an inverse power law distribution as in the original form of SOC. This new phenomenon is herein labeled self-organized temporal criticality (SOTC). We compare this bottom-up self-organization process to the adoption of a global choice rule based on assigning to all the units the same value *K*, with the time evolution of common *K* being determined by consciousness of the social benefit, a top-down process implying the action of a leader. In this case self-organization is impeded by large intensity fluctuations and the global social benefit turns out to be much weaker. We conclude that the SOTC model fits the requests of a manifesto recently proposed by a number of European social scientists.

## 1. Introduction

One of the main goals of computational social models is to quantify the mechanisms generating the emergence of collective behavior of social groups. A particularly useful modeling tool in this regard has been evolutionary game theory. This tool was used to explain the emergence and survival of cooperation in society, in contrast to the widely recognized selfish character of single individuals. Axelrod and Hamilton (1981) have addressed the apparent contradiction and their work has drawn the attention of an increasing number of researchers to the surprising condition that altruism may have originated much earlier than the dawn of human civilization. Altruism may, in fact, correspond to the birth of life itself, although the concepts of kinship and reciprocity, widely adopted in game theory, seem to refer to complex social networks and not to individuals. In fact, Axelrod and Hamilton based their life evolution study on the use of the Prisoner's Dilemma game, with its crucial conflict between the individual's temptation to cheat and to act in the community's benefit, a model that seems to apply only to human society.

More recently, evolutionary game theory concepts, which were apparently introduced to discuss the social effect of public good, are used to gain insight into enzyme chemistry processes (Archetti and and Scheuring, 2016). Another sociological concept, currently adopted to illustrate the conflict between the use of limited shared resources and individual self-interest (Hauser et al., 2014), “the tragedy of the commons”, has been used (Stewart and Plotkin, 2016) to discuss the evolution of cooperation in ecological networks.

The argument of network reciprocity, in the form illustrated by Nowak and May (1992), rests on the observation that in a network of cooperators and defectors, the richer environment of cooperators prevents the spreading of defectors. This argument has been questioned by some, noting the social activities in which the individuals are engaged, who are also involved in playing the Prisoner's Dilemma game. The additional social interaction of the individuals within this social group was found to disrupt network reciprocity (Vilone et al., 2012, 2014). However, when this additional activity is based on individuals imitating the choices made by their nearest neighbors, it may favor the survival of cooperation (Mahmoodi and Grigolini, 2017). This survivability is a consequence of the imitation strength being sufficiently strong to generate criticality as in the Decision Making Model (DMM) (West et al., 2014).

The criticality condition exploited by Mahmoodi and Grigolini (2017) is obtained by tuning the imitation strength to the theoretical value that in the limiting case of an infinitely large network is expected to be determined by an Ising-like prescription, since the DMM used is in the Ising universality class (West et al., 2014). Criticality entails long-range correlation among the members of the society, even those communicating solely by means of nearest-neighbor interactions. Such criticality has been interpreted as a form of global intelligence, identified as *swarm intelligence* (Vanni et al., 2011), a phenomenon that may be shared by microbial communities and mechanisms of carcinogenesis (Rosenfeld, 2013), as well as, by neural systems (Hesse and Gross, 2014). In the specific case of individuals playing the Prisoner's Dilemma game, the criticality-induced swarm intelligence enables the members of society to become aware of the benefits of network reciprocity, and thereby biases their interactions to favor, rather than disrupt, this network property (Mahmoodi and Grigolini, 2017).

The manifesto of computational social science (Conte et al., 2012) relies on the assumption that criticality is a consequence of self-organization, and thereby implies that social criticality is a form of self-organized criticality (SOC). A word of caution is appropriate here, now that the term SOC has been used. A 25-year review of the concepts and controversies surrounding SOC (Watkins et al., 2016), emphasize that SOC occurs in open, extended, dissipative dynamical systems that automatically go to the critical state. This is distinct from a continuous phase transition where *at a critical point* correlations become long-range and are characterized by an inverse power-law (IPL) probability density function (PDF). In order to arrive at the critical point an external control parameter, such as temperature, must be fine-tuned to its critical value. We refer to that control parameter with the symbol *K*. On the other hand, SOC occurs universally where any fine-tuning is accomplished by means of its internal dynamics (Bak and Chen, 1989). This independence from an external tuning is the defining property of a SOC phenomenon.

The emergence of SOC is usually signaled by the births of anomalous avalanches, see Zapperi et al. (1995); Martinello et al. (2017) for more recent work. In the present paper we illustrate a form of SOC based on the spontaneous search for the critical value of the parameter *K*, which is selected by the network through a *bottom up* process, that is, through the dynamic behavior of the individuals and is not externally imposed. The main signature of self-organized criticality of this paper is the time interval between two crucial events, with a non-exponential waiting time probability distribution density (PDF), a property referred to as *temporal complexity* in earlier work (Turalska et al., 2011). *We therefore refer to the form of SOC developed in this paper as self-organized temporal criticality (SOTC)*. The crucial events are defined by comparing the variable *K*(*t*) to its time average $\bar{K}$ and are identified with the variable $\zeta \left(t\right)=K\left(t\right)-\bar{K}$ changing sign.

The only earlier form of SOC known to us yielding temporal complexity is Lipiello et al. (2005), showing that SOC of Bak and Chen (1989) has no temporal complexity, insofar as the time duration to reach equilibrium in that case is described by an exponential waiting time PDF. We do not rule out the emergence of the traditional SOC avalanches, but we leave it as subject for future research work.

We emphasize that the form of SOTC is realized in full accordance with the spirit of the Axelrod and Hamilton (1981) theoretical perspective. In fact the payoff of the choices made by the individuals of the composite network is established using the Prisoner's Dilemma game, without neglecting the incentive to defection. The choice of the strategy to adopt is determined by the individual's imitation of the choices made by their nearest neighbors. The single units only decide to increase or decrease their tendency to imitate these choices according to whether on the basis of the last two payoffs this imitation increased or decreased the benefit to them as an individual. This indirect and apparently blind strategy choice does not disrupt the beneficial effects of network reciprocity (Nowak and May, 1992), but it is a way of efficiently establishing the reciprocity condition hypothesized by Axelrod and Hamilton (1981).

Returning to the SOTC issue, we stress that the imitation strength *K* is not a conventional fine tuned control parameter, that is artificially fixed to make the network achieve criticality. The parameter *K* is freely selected by the dynamics of the network itself.

The numerical calculations presented herein show that increasing the dependence of the individuals on the strategic choices of their neighbors has the effect of increasing their payoff. Imitation of the choices of their neighbors is a form of social interaction that is made at the level of the individuals and is not forced upon them in a top-down process. There exists a parameter, call it χ, which determines the rate of change of *K*, as a function of the last two payoffs. However, no recourse is made to the fine tuning of this parameter, insofar as changing χ has only the effect of influencing the time scale of the process of transition to altruism. This is, as we show, a bottom-up process that generates self-organization, and along with self-organization generates swarm intelligence, with the ultimate effect of increasing the wealth of society, thereby affording strong support to the increasing conviction that real social improvements do not require the action of benevolent dictators (Helbing and Pournaras, 2015; Helbing, 2017).

## 2. The prisoner's dilemma game

This Section is devoted to illustrating the criteria adopted in the subnetwork of logical choices to evaluate the payoff associated to the cooperation or the defection choice. This is done using the Prisonner's Dilemma game. This game was originally introduced as a metaphor for the problems affecting the emergence of cooperation (Axelrod and Hamilton, 1981). Two players interact and receive a payoff from their interaction adopting either the defection or the cooperation strategy. If both players select the cooperation strategies, each of them gets the payoff *R* and their society receives the payoff 2*R*. The player choosing the defection strategy receives the payoff *T*. The temptation to cheat is established by setting the condition

(1) $$\begin{array}{r} T>R. \end{array}$$

However, this larger payoff is assigned to the defector only if the other player selects cooperation. The player selecting cooperation receives the payoff *S*, which is smaller than *R*. If the other player also selects defection, the payoff for both players is *P*, which is smaller than *R*. The game is based on the crucial inequalities

(2) $$\begin{array}{r} T>R>P>S. \end{array}$$

It is evident that for a player, let us call her #1, the choice of defection condition is always the most convenient, regardless of the choice made by the other player, let us call her #2. In fact, if the player #2 selects cooperation, player #1 receives *R*, but the better payoff *T* if she selects defection. If player #2 selects defection, player #1 receives the payoff *S* if she selects cooperation and the larger payoff *P* if she selects defection. However, the whole society receives the largest payoff, 2*R*, if both players select cooperation, a smaller payoff, *T* + *S*, if one selects defection and the other cooperation, and the smallest payoff, 2*P*, if both players select defection.

Axelrod and Hamilton (1981) noted that if the Prisoner's Dilemma game is played only once no strategy can defeat the strategy of pure defection. If the game is played more than once, reciprocity may make the choice of cooperation become the winning strategy. Nowak and May (1992) substantiated this concept with their model of network reciprocity. The players are the nodes of a regular two-dimensional lattice and each players can interact with her nearest neighbors. The players are initially randomly assigned either the cooperation or the defection strategy. After each play, before the next play, they are left free to update their strategy selecting the strategy of their most successful nearest neighbor. Since the environment of the cooperators, as above noted, is wealthier than the environment of defector, it is possible that the most successful nearest neighbor is a cooperator, rather than a defector. This is a rational form of imitation that may lead to the survival of cooperators. In this paper we use only the Prisoner's Dilemma game to evaluate the payoff and we realize the network reciprocity with the interaction between the two subnetworks that will be described in Section 4.

## 3. Decision making model

In this Section we illustrate the dynamics of the subnetwork where decisions are made by the individuals under the influence of their nearest neighbors. These dynamics are realized by using the *Decision Making Model* (DMM) (West et al., 2014). In the earlier work (Mahmoodi and Grigolini, 2017), this model was denoted as Local Conformism Model (LCM), to emphasize that according to the work of Vilone (Vilone et al., 2012, 2014) social influence may disrupt the benefits of the Nowak and May network reciprocity (Nowak and May, 1992), if the social influence does not establish a correlation between the dynamics of different individuals. As we shall see in Section 4, the interaction between the DMM subnetwork and the Prisoner's dilemma subnetwork generates criticality. The individuals of the composite networks in this and in the following sections of this paper are the nodes of a regular two dimensional network, denoted by the symbol *r* equivalent to the double index (*i, j*).

Here we describe the DMM behavior in the absence of this interaction. The transition rate from cooperation to defection, $g_{CD}^{\left(r\right)}$, is given by

(3) $$\begin{array}{r} g_{CD}^{\left(r\right)}=g_{0}exp\left[-K\left(\frac{J_{C}^{\left(r\right)}-J_{D}^{\left(r\right)}}{J}\right)\right] \end{array}$$

and the transition rate from defection to cooperation, *g*_*DC*_, is given by

(4) $$\begin{array}{r} g_{DC}^{\left(r\right)}=g_{0}exp\left[K\left(\frac{J_{C}^{\left(r\right)}-J_{D}^{\left(r\right)}}{J}\right)\right]. \end{array}$$

The meaning of this prescription is as follows. The parameter 1/*g*_0_ defines the time scale of interest and we set *g*_0_ = 0.01 throughout this paper. Time is discrete, starting from 1 and the distance between two consecutive time events is Δ*t*, which is also selected to be 1. We consider *M* = *N* × *N* individuals of a regular two-dimensional network with periodic boundary condition. Each individual has *J* neighbors (four in the case of the regular two-dimensional lattice used herein). $J_{C}^{\left(r\right)}$ neighbors are in the cooperation state and $J_{D}^{\left(r\right)}$ of them are in the defection state. If the individual *r* is in the cooperation state *C*, and the majority of its neighbors are in the same state, then the transition rate becomes smaller and the individual sojourns in the cooperation state for a longer time. If the majority of its neighbors are in the defection state *D*, then the individual *r* sojourns in the cooperator state for a shorter time. An analogous prescription is used if the individual *r* is in the defection state.

To denote the effect of imitation we assign to the units selecting the cooperation state the value ξ_*r*_ = 1 and to the units in the defection state the value ξ_*r*_ = −1. To establish whether cooperation or defection is selected by the social system we use the mean field *x*(*t*) defined by

(5) $$\begin{array}{r} x\left(t\right)=\frac{1}{M}\overset{M}{\underset{r}{\sum }}\xi_{r}. \end{array}$$

For *K* < *K*_*C*_ the mean field vanishes, but at criticality, when *K* = *K*_*C*_, the social system can select either the cooperation or the defection branch yielding for *K* ≫ *K*_*C*_ either the value *x* = 1 or *x* = −1. The critical value of the control parameter *K* is *K*_*C*_ = 1 in the all-to-all coupling case and *K*_*C*_ = 1.5 (M = 100) in the case of a regular two-dimensional lattice (Mahmoodi and Grigolini, 2017).

## 4. Self-organization

The earlier work (Mahmoodi and Grigolini, 2017) was based on the assumption that the players are the nodes of a regular two-dimensional network. The players adopt for most of their time the blind imitation of LCM and for a small portion of their time the rational imitation of Nowak and May. Quite surprisingly the exceedingly large use of the blind imitation, rather than disrupting the benefits of network reciprocity, has the effect of forcing the system to select the cooperation branch, leading to the extinction of defectors. This is an interesting effect that is due however to the fine tuning of LCM imitation strength to the critical value generating criticality.

The main purpose of the present paper is to overcome this limitation with a natural SOC process. This significant step ahead is realized without using the Nowak and May network reciprocity. The earlier work (Mahmoodi and Grigolini, 2017) was based on the single units adopting of the Nowak and May network reciprocity for a limited amount of their time and on the criticality-induced swarm intelligence making the network realize the benefits of Nowak and May network reciprocity. Herein the swarm intelligence condition emerges from self organization, which makes it possible for the collective mind to realize that the choice of cooperation makes society wealthier.

We adopt the choice of parameters made by Gintis (2014) and set *R* = 1, *P* = 0, *T* − *R* = 0.5 and *S* = 0. We evaluate the social benefit for the single individual and for the community as a whole as follows. We define first the payoff *P*_*r*_ of the single unit *r*. Each unit gets a total payoff from the play with its four nearest neighbors. Namely we have to consider four pairs of players. If both players of a pair are cooperators the contribution to the payoff of the unit *r* is *B*_*r*_ = 2. If one of the two playing units is a cooperator and the other is a defector, the contribution to the payoff of the unit *r* is *B*_*r*_ = *T*. If both players are defectors the contribution to the payoff of the unit *r* is *B*_*r*_ = 0. The payoff *P*_*r*_ of the unit *r* is the sum over the four *B*_*r*_. The mean benefit for the units of this society is

(6) $$\begin{array}{r} \Pi =\frac{1}{M}\overset{M}{\underset{r}{\sum }}P_{r}. \end{array}$$

Self-induced criticality is realized in two distinct ways: *individual* and *global*:

### 4.1. Individual

It is important to notice that *K*_*r*_, the value of imitation strength adopted by the generic unit *r* to pay attention to the choices made by its four nearest neighbors about selecting either the cooperation or the defection strategy, is not necessarily adopted by its four nearest neighbors. In other words, the imitation strength *K*_*r*_(*t*) is unidirectional and it goes from *r* to all its nearest neighbors. The imitation strength *K*_*r*_(*t*) changes from individual to individual, as well as in time, and it is consequently very different from the control parameter *K* of the conventional DMM phase transition processes, where *K* has a single value throughout the whole network.

Each member is assigned a vanishing initial imitation strength, corresponding to a total independence of the choices made by its nearest neighbors. At each time step the units play the game and they independently change their imitation strength doing the implicit assumption that the increase (decrease) of their individual payoff in the last two trades makes convenient for them to increase (decrease) the imitation strength. More precisely, they adopt the following rule. As stated earlier, time is discrete and the interval between two consecutive time events is Δ*t* = 1. The imitation strength of the unit (*i, j*) changes in time according the individual choice rule:

(7) $$\begin{array}{r} K_{r}\left(t\right)=K_{r}\left(t-\Delta t\right)+\chi \frac{\left(P_{r}\left(t-\Delta t\right)-P_{r}\left(t-2\Delta t\right)\right)}{\left(P_{r}\left(t-\Delta t\right)+P_{r}\left(t-2\Delta t\right)\right)}, \end{array}$$

where the parameter χ determines the intensity of the interest of the units for their payoff. *P*_*r*_(*t*) is the payoff of the unit *r* at time *t*. The intensity of the imitation strength increases or decreases according to whether in the two last trades the individual payoff increases or decreases. If the payoff does not change, the imitation strength remains unchanged. To make a comparison with the global condition we evaluate also the mean imitation strength

(8) $$\begin{array}{r} K\left(t\right)=\frac{1}{M}\overset{M}{\underset{r}{\sum }}K_{r}\left(t\right). \end{array}$$

**Figure 1:**
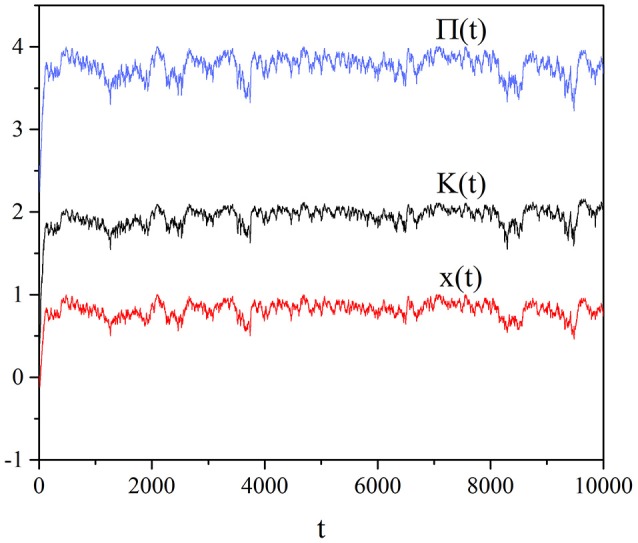
Individual case: Time evolution of, from the top to the bottom, the benefit Π(*t*) of Equation (6), the variable *K*(*t*) of Equation (8) and the mean field *x*(*t*) of Equation (5). We adopted the values: *T* = 1.5, χ = 4, *M* = 100.

Figure 1 shows the self-organization of the social system as a result of individual choices of the interacting units. The average imitation strength moves very quickly from the vanishing initial value, corresponding to no social interaction, toward a maximal value which is *K* ≈ 1.8. Notice that in the absence of interaction with the Prisoner's Dilemma process, the Ising-like DMM for the case of a regular two-dimensional lattice (West et al., 2014) would require the critical value *K*_*C*_ ≈ 1.65 for *M* = ∞ and, as earlier mentioned, *K*_*C*_ ≈ 1.5 for *M* = 100.

It is important to notice that in the case of criticality generated by a fine tuning parameter the fluctuations of the mean field around the equilibrium value have an increasing intensity upon decrease of the number of units (Beig et al., 2015). We show that this property is shared by the SOTC. Let us define

(9) $$\begin{array}{r} \zeta \left(t\right)=K\left(t\right)-\bar{K}, \end{array}$$

(10) $$\begin{array}{r} \zeta \left(t\right)=x\left(t\right)-\bar{x} \end{array}$$

and

(11) $$\begin{array}{r} \zeta \left(t\right)=\Pi \left(t\right)-\bar{\Pi }. \end{array}$$

The symbols $\bar{K}$, $\bar{x}$ and t $\bar{\Pi }$ denote the time mean values of the corresponding fluctuations evaluated on the time series of length *L*. The intensity of these fluctuations is defined by

(12) $$\begin{array}{r} \Delta \zeta =\sqrt{V\left(\zeta \right)}, \end{array}$$

where

(13) $$\begin{array}{r} V\left(\zeta \right)\equiv \frac{\int_{0}^{L}dt\zeta {\left(t\right)}^{2}}{L}, \end{array}$$

with *L* denoting the length of time series.

We expect that

(14) $$\begin{array}{r} \Delta \zeta \propto \frac{1}{M^{\nu }}. \end{array}$$

**Figure 2:**
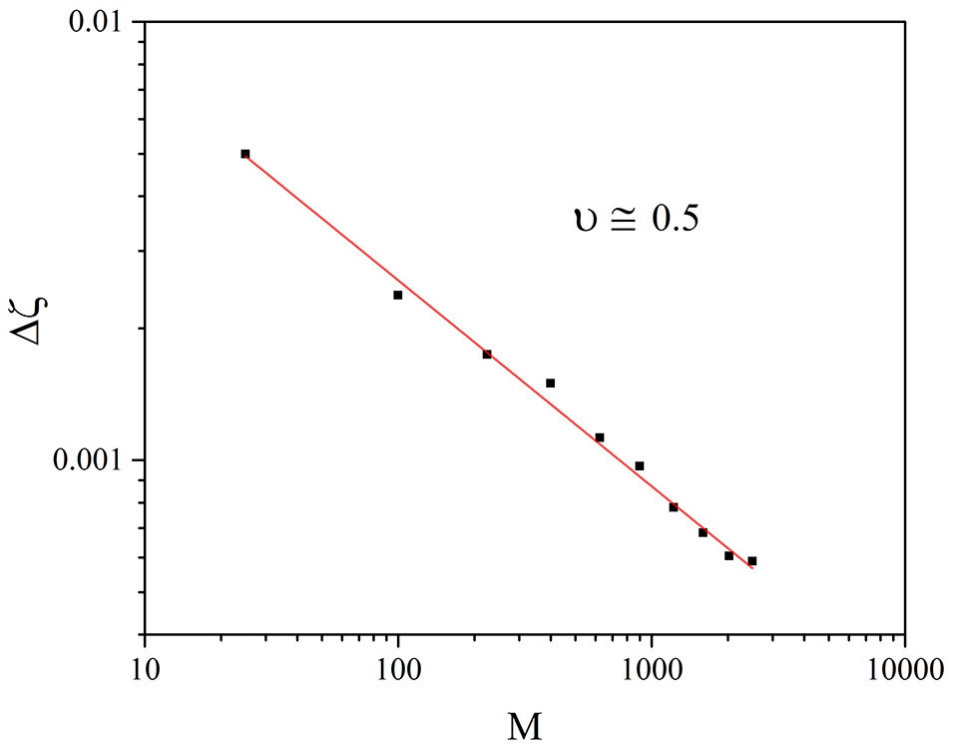
Individual case: The square root of the fluctuation variance, Δζ of Equation (12), as a function of *M*. In this case $\zeta \equiv K\left(t\right)-\bar{K}$. We adopted the values: *T* = 1.5, χ = 4.

In the case of the criticality with a fine tuning parameter of Beig et al. (2015), ν = 0.25. Presently we do not have a theory to determine ν for SOTC, but it is interesting to notice that the numerical calculations illustrated in Figure 2 show that ν = 0.5, making fluctuation intensity of ζ(*t*) more significant than in the case of the ordinary criticality of Beig et al. (2015). The fluctuations of ζ are determined by the crucial events and their complexity constitutes the information transferred from one to another self-organizing network. Increasing the intensity of these fluctuation favors this transport process, but, as we see in Section 5, there exists a crucial value of *M*, below which no signs of the IPL properties of temporal complexity remain.

### 4.2. Global

In the global case we assume that all the units share the same *K*, which changes in time according global choice rule:

(15) $$\begin{array}{r} K\left(t\right)=K\left(t-\Delta t\right)+\chi \frac{\left(\Pi \left(t-\Delta t\right)-\Pi \left(t-2\Delta t\right)\right)}{\left(\Pi \left(t-\Delta t\right)+\Pi \left(t-2\Delta t\right)\right)}. \end{array}$$

The global payoff Π(*t*) is evaluated by making a sum over all possible pairs (*i, j*), as defined by Equation (6). In the global case we select as initial condition *K*(0) = 0.5. The implicit rationale for Equation (15) is that the social community makes the same assumption as the individuals of Equation (7), namely that a payoff increase (decrease) in the last two trades before setting the imitation strength to adopt at time *t* suggests its increase (decrease) to be convenient. This condition requires a top down process, a decision made by a leader on the appropriate imitation strength that the single units are forced to adopt for the benefit of society.

**Figure 3:**
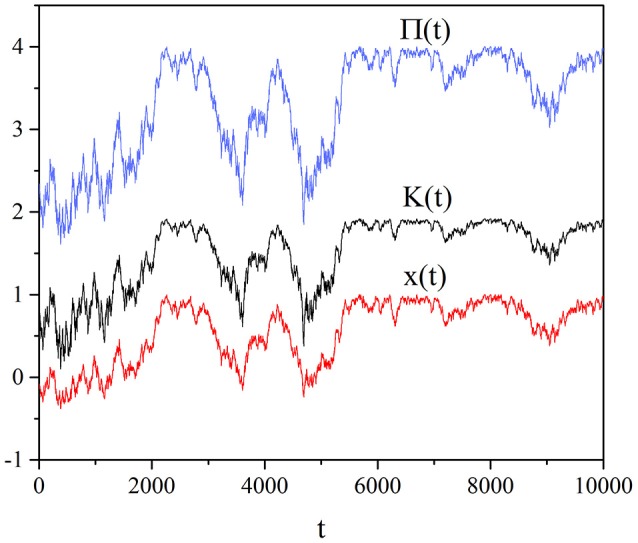
Global case: Time evolution of, from the top to the bottom, the benefit Π(*t*) of Equation (6), the variable *K*(*t*) of Equation (15) and the mean field *x*(*t*) of Equation (5). We adopted the values: *T* = 1.5, χ = 4, *M* = 100.

Figure 3 shows the self-organization of the social system as a result of the global choices with all units sharing the same value of imitation strength. The qualitative behavior is similar to that of the individual choice, thereby suggesting that the individual choices of the interacting units are characterized by the same intelligence as that of the leader driving the global choice. In fact, the global case is tacitly based on the assumption that the collective payoff is communicated to the individuals who are forced to share the same imitation strength, while the individual choice is based on the realistic assumption that each unit is aware of its individual payoff, without requiring any information transmission from a leader to the individuals. Thus, we are led to the conclusion that the SOTC should be interpreted as a spontaneous emergence of the swarm intelligence that in the earlier work is based on tuning a control parameter *K* to a critical value (Vanni et al., 2011).

The comparison between Figure 3 and Figure 1 leads us to an even more interesting observation. We notice that the global choice yields an intermittent behavior that has the effect of significantly reducing the social benefit, even if, in qualitative accordance with the individual choice rule, the system moves toward cooperation. The individual choice rule is more efficient than the global choice rule and is not affected by the strong fluctuations that intermittently reduce the social wealth. For this reason we are inclined to identify the society leader of the global choice with the benevolent dictator discussed by Helbing and Pournaras (2015). According to these authors, in fact, the centralized top-down organization has various flaws reducing their efficiency and they propose instead a bottom up pluralistic model inspired by neural processes. We believe that the numerical results of this paper lend support to the conclusion that the bottom up process of the individual choice is more efficient than the top down process of the global choice. Therefore it seems that our model of a self-organizing network supports the concluding remarks of Helbing: “I am convinced that co-creation, co-evolution, collective intelligence, self-organization and self-governance, considering externalities (i.e., external effects of our actions), will be the success principles of the future” (Helbing, 2017). In fact, the spontaneous transition to criticality proposed in this paper is associated with the emergence of significant resilience and adaptivity. This will be made clear in the next two sections devoted to designate temporal complexity rather than spatial avalanches as a signature of criticality (Section 5) and to illustrate the related property of complexity matching (Section 6). We think that the individual choice is an example of SOTC which is more interesting than the global choice and for this reason we restrict our attention to study the individual dependence on *M*.

## 5. Temporal complexity

How is criticality defined in a social model? This is a difficult question, because even in the well known condition of the Ising Universality class (West et al., 2014) we have to take into account the observation of systems with a number of units much smaller than the virtually infinite Avogadro number of units in a physical network, which has the effect of breaking the singularity condition of ordinary thermodynamic systems. The authors of Turalska et al. (2011) and Zare and Grigolini (2013) defined the occurrence of criticality through the observation of *temporal complexity*. In the case of a phase transition falling in the range of the Ising Universality class, the occurrence of phase transition in a system with a finite number of interacting units, at criticality the mean field *x*(*t*) fluctuates around the vanishing value and the time interval between two consecutive origin crossings is described by a markedly non-exponential waiting time PDF ψ(τ) (Turalska et al., 2011). In the subcritical regime the interval between two consecutive crossings of the origin is exponential and in the supercritical regime the interval between two consecutive crossings of the non-vanishing mean field is again exponential. Temporal complexity emerges at criticality and for the proper function of the network it requires that the IPL PDF of the distances between two consecutive crucial events is exponentially truncated (Lukovic and Grigolini, 2008; Vanni et al., 2011; Beig et al., 2015).

The adoption of temporal complexity as the signal of criticality occurrence led the authors of Zare and Grigolini (2013) to notice that this may be a more convenient indicator than the observation of avalanches with a PDF becoming IPL. This assumption was confirmed by the authors of Mafahim et al. (2015), who found that two networks in critical states signaled by temporal complexity exchange information with an efficiency larger than in the correspondence with the state of criticality signaled by IPL avalanches. The reason for the close connection between maximal efficiency of information transport and temporal complexity is based on the theory illustrated in Vanni et al. (2011), Turalska et al. (2011) and Luković et al. (2014). Criticality generates non-Poisson renewal events characterized by the IPL indexes and the exchange of information is based on the occurrence of the non-Poisson renewal events of network influencing the occurrence of the non-Poisson renewal events of the other network, this being the Principle of Complexity Management (West et al., 2014).

We conjecture that the SOTC model spontaneously generates temporal complexity. The present section is devoted to establishing that this conjecture is correct and to prove it we use a numerical approach treatment, applied to the individual choice rule.

**Figure 4:**
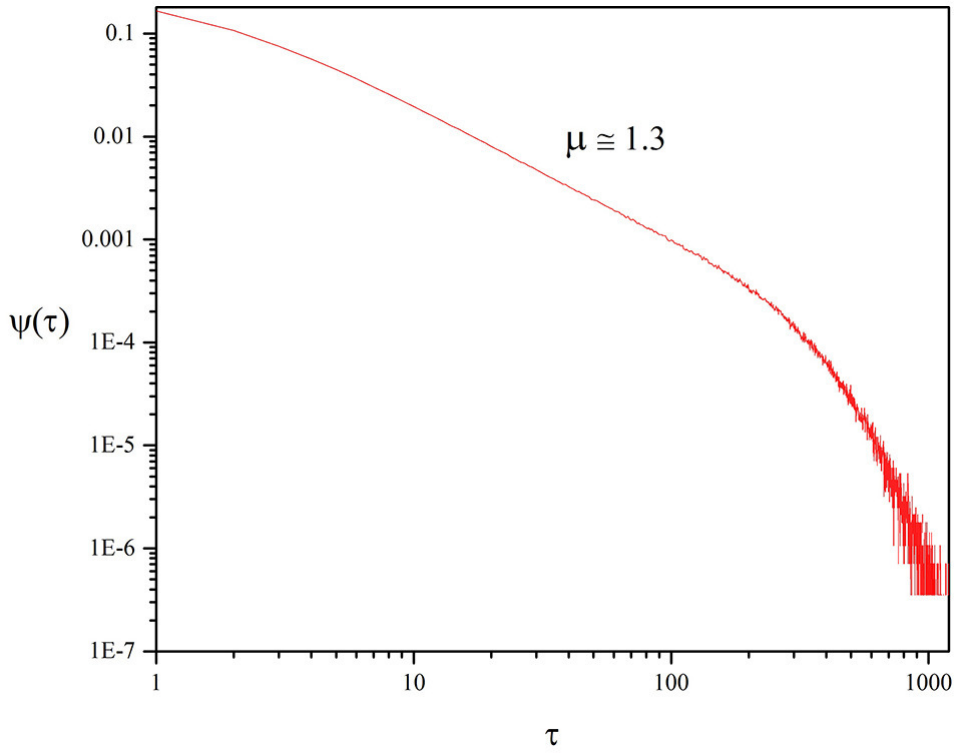
Waiting time distribution density of the time distance between two consecutive origin crossings of the function ζ(*t*) defined by Equation (10). We adopted the values: *T* = 1.5, χ = 4, *M* = 100.

We monitor the times at which the fluctuations ζ(*t*) cross the origin and find that the three waiting time PDF coincide. For simplicity, in Figure 4 we illustrate only the waiting time PDF of ζ(*t*) of Equation (10). The fact that fluctuations of *K*(*t*), *x*(*t*), and Π(*t*) around their average values yield indistinguishable results is an incontrovertible consequence of the fact that all three properties are driven by the non-Poisson renewal events with the same statistical properties.

It is known that in systems of finite size the IPL are exponentially truncated (Beig et al., 2015). As a consequence, the non-Poisson nature of the crucial events is established analyzing the intermediate time region. Therefore, to estimate with accuracy the IPL index generated by the SOTC of Section 3 we focus on the time region between *t* ≈ 2 and *t* ≈ 200, as illustrated by Figure 4. We find that the waiting time PDF is IPL:

(16) $$\begin{array}{r} \psi \left(\tau \right)\propto \frac{1}{\tau^{\mu }} \end{array}$$

with

(17) $$\begin{array}{r} \mu =1.3. \end{array}$$

rather than the traditional μ = 1.5 generated by DMM at criticality (Beig et al., 2015).

**Figure 5:**
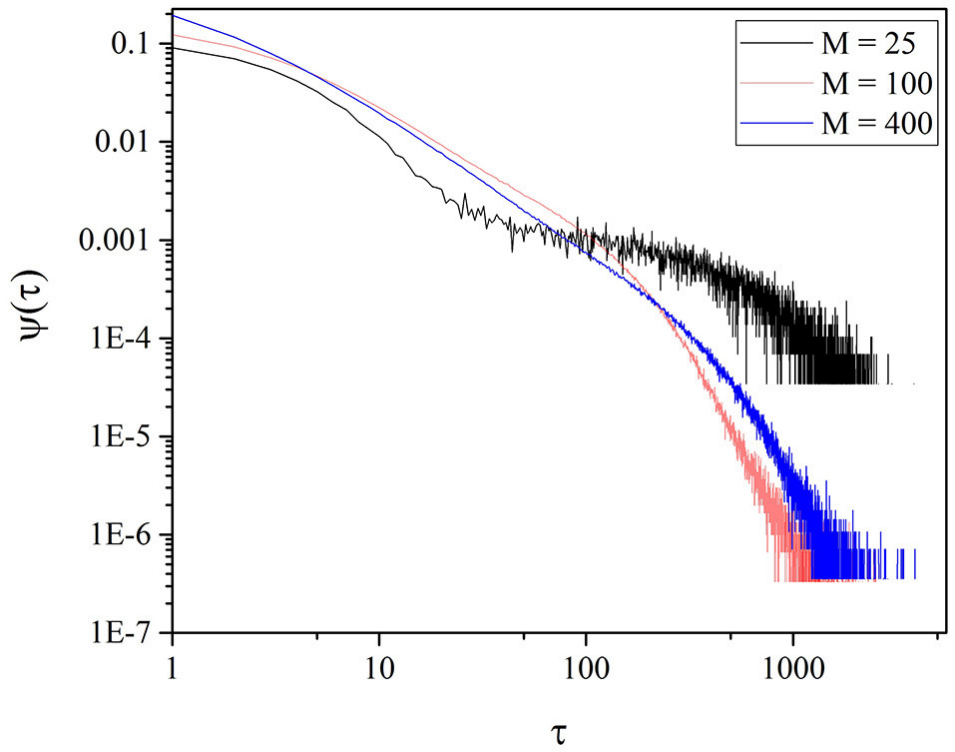
Waiting time PDF of the time distance between two consecutive origin crossings of the function ζ(*t*) defined by Equation (10) for different values of *M*. We adopted the values: *T* = 1.5, χ = 4.

It is interesting to notice the length of the time region characterized by μ = 1.3 depends on *M*. Figure 5 shows that for *M* = 225 the IPL region is more extended. We also see, Figure 5, that for *M* = 25 the short time region is characterize by a very large value of μ and by a pronounced exponential shoulder, both conditions generating non-crucial events. Although the fluctuation intensity is very large, much larger than for *M* = 100 and *M* = 225 (see Figure 2), the extended IPL region is lost and with it the efficiency of the process of information transport, as we see in Section 6.

It is out of scope of this paper to afford a theory for these results, but to help the reader to appreciate the importance of the SOTC model we mention that the research work done some years ago (Failla et al., 2004) on the random growth of surfaces, which can be interpreted as a form of SOC (Kim et al., 1992), suggests that the Laplace transform of the survival probability

(18) $$\begin{array}{r} \Psi \left(t\right)\equiv \int_{t}^{\infty }dt^{\prime }\psi \left(t^{\prime }\right) \end{array}$$

has the following form, using the notation $\hat{\Psi }\left(u\right)\equiv \int_{0}^{\infty }dtexp\left(-ut\right)\Psi \left(t\right)$,

(19) $$\begin{array}{r} \hat{\Psi }\left(u\right)=\frac{1}{u+\lambda^{\alpha }{\left(u+\Delta \right)}^{1-\alpha }}, \end{array}$$

where α = μ − 1 < 1 and λ is a parameter measuring the interaction between the unit and Δ ∝ λ determines the exponential truncation of ψ(*t*). In the case where λ ≫ Δ an extended time interval exists, 1/λ ≪ *t* ≪ 1/Δ, where $\psi \propto \frac{1}{t^{1+\alpha }}$, thereby yielding Equations (16, 17) when α = 0.3. This structure is lost for *M* = 25, when temporal complexity is gone.

The most important reason for the use of Equation (19) is that when an extended IPL emerges from it, the process is distinctly non-ergodic. The spectrum of the fluctuation in that case cannot be derived from the Wiener-Khintchine theorem, resting on the stationarity assumption. It is necessary to take into account that μ < 2, μ = 1.3 in this case, the average time interval between two consecutive events diverges, thereby making non-stationary the process driven by the crucial events. This anomalous condition yields (Lukovic and Grigolini, 2008)

(20) $$\begin{array}{r} S\left(\omega \right)\propto \frac{1}{L^{2-\mu }}\frac{1}{\omega^{\beta }}, \end{array}$$

with

(21) $$\begin{array}{r} \beta =3-\mu . \end{array}$$

**Figure 6:**
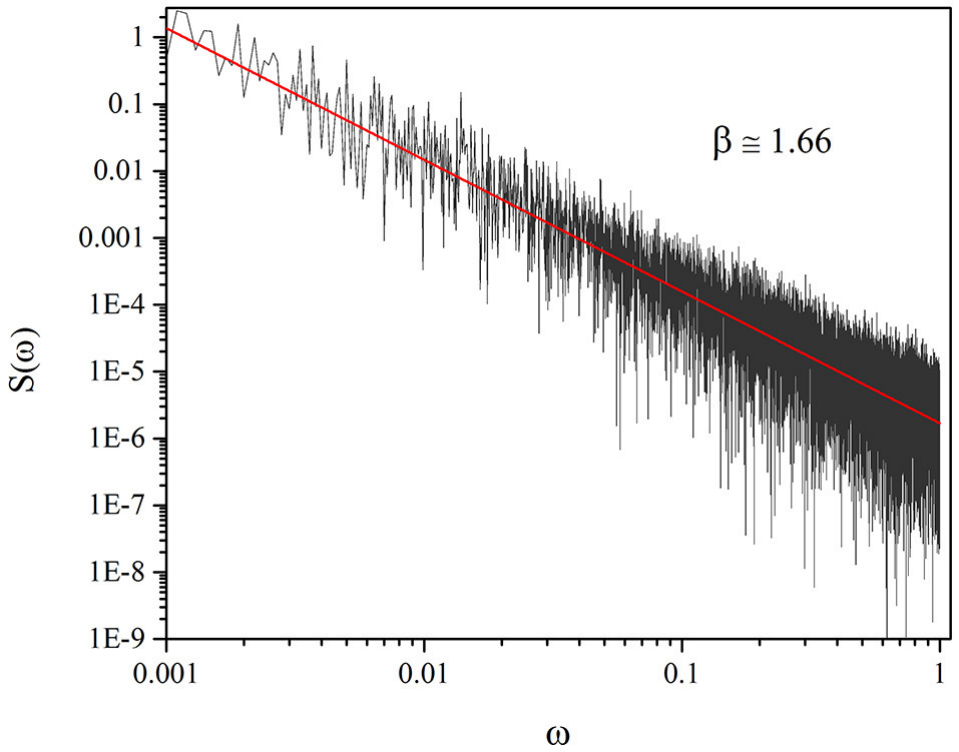
Spectrum of the fluctuations of *K*(*t*) for *T* = 1.5, χ = 4, *M* = 100.

In the case where the process yields a slow but stationary correlation function, we would have β < 1 (Lukovic and Grigolini, 2008). Evaluating the power spectrum in this case becomes computationally challenging because, as shown by Equation (20), the noise intensity decreases with increasing the length *L* of the time series. Nevertheless, the results of Figure 6, yielding β = 1.67, afford a satisfactory support to our claim that the origin crossings of ζ are renewal non-stationary events. In conclusion, the SOTC spontaneously generates the crucial events of criticality-induced temporal complexity.

## 6. Complexity matching

**Figure 7:**
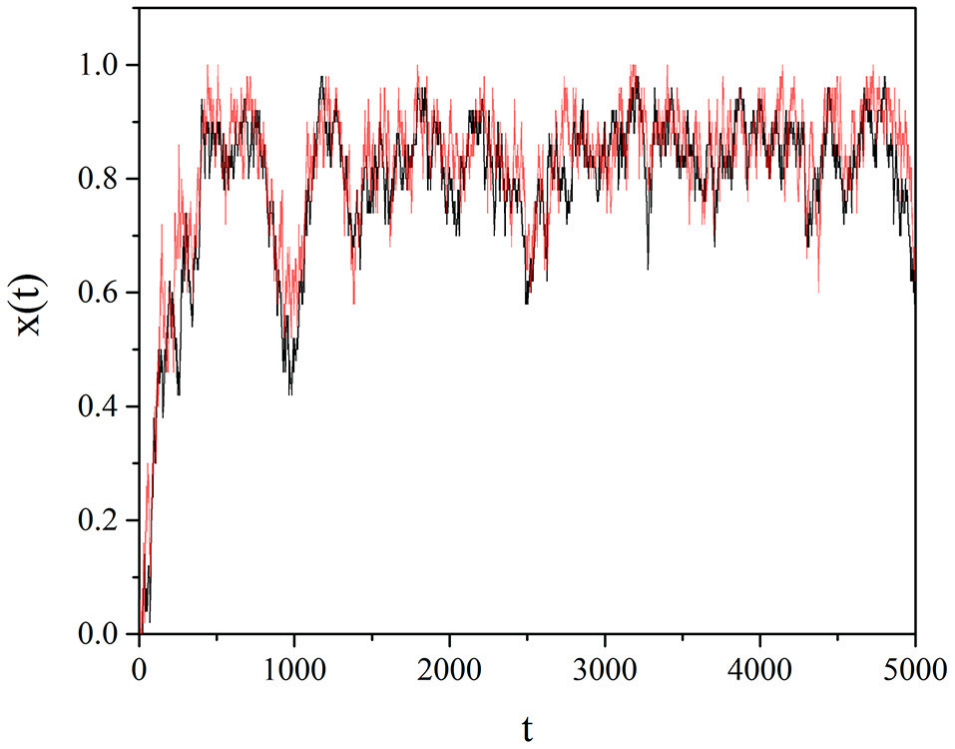
The mean field *x*(*t*) of two identical self-organizing networks connected to each other according to text illustration. The self-organization is realized through the individual choice. We adopted the values: *T* = 1.5, χ = 4, *M* = 100.

It has to be stressed that the synchronization between two networks is not a form of chaos synchronization. It is due to the non-Poisson renewal events of the driving network exerting influence on the renewal events of the driven network, as pointed out in Piccinini et al. (2016) (see also Aquino et al., 2010). The non-Poisson renewal events are generated by criticality and in the composite network proposed in this paper they are the result of a spontaneous process. In Figure 7 we illustrate the remarkable synchronization between two identical self-organized complex networks, *A* and *B*, with *M* = 100. We select a random subgroup *S*_*A*_ of the network *A*, consisting of 5% of the units of *A*, and we assign to each of them the strategy of a unit of *B*, also randomly selected. We follow the same prescription with a subgroup *S*_*B*_ consisting of 5% of units of *B* following the strategy of randomly selected units of *A*. We see in Figure 7 that a remarkable synchronization between the two networks is realized.

To establish the accuracy of this synchronization we apply the same procedure to two self-organized networks *A* and *B* with *M* changing from *M* = 25 to *M* = 900. We study the cross correlation *C*(τ) defined by

(22) $$\begin{array}{r} C\left(\tau \right)\equiv \frac{\int_{0}^{L-\tau }dt\left(x\left(t\right)-\bar{x}\right)\left(y\left(t+\tau \right)-\bar{y}\right)}{\sqrt{\int_{0}^{L}dt{\left(x\left(t\right)-\bar{x}\right)}^{2}\int_{0}^{L}dt{\left(y\left(t\right)-\bar{y}\right)}^{2}}}. \end{array}$$

**Figure 8:**
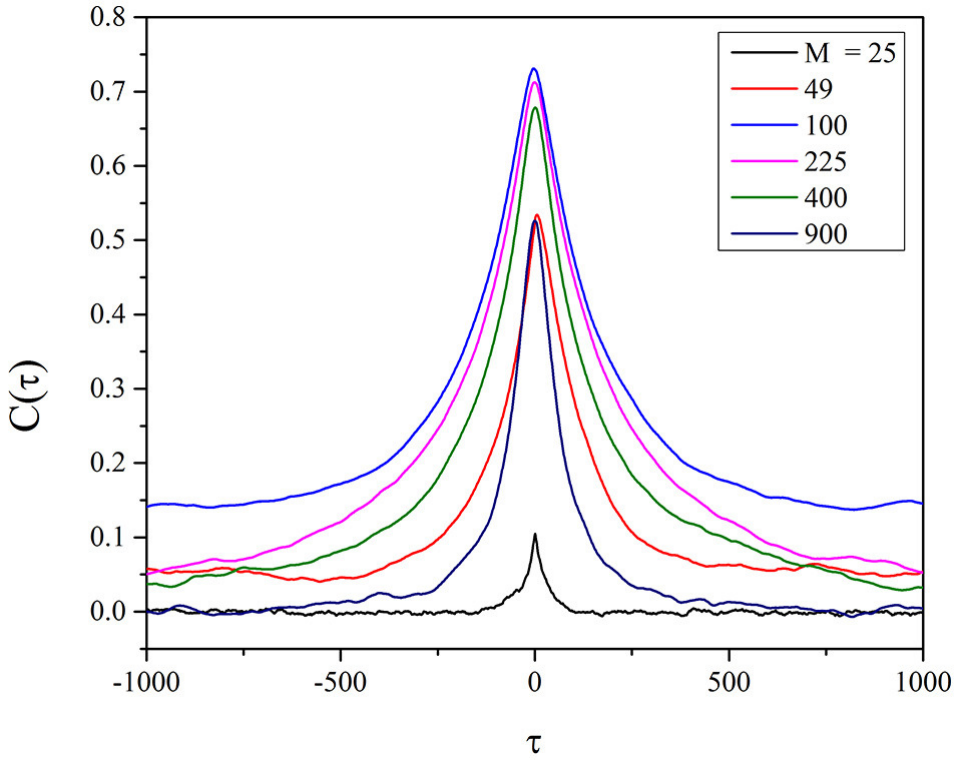
Cross correlation between two identical self-organizing networks. The different curves refer from top to bottom to: *M* = 100, 225, 400, 49, 900, 25. The self-organization is realized through the individual choice. We adopt for the cross-correlation the definition of Equation (22) and the values: *T* = 1.5, χ = 4.

The numerical result is illustrated in Figure 8. To understand the importance of this result, we must make a short digression to mention an important result recently reached in the field of evolutionary game theory (Stewart and Plotkin, 2016). This earlier paper stresses the connection between emergence of cooperation and memory. Our SOTC model based on the memory of the last two trades before making a decision about the degree of attention to the nearest neighbor may be related to the model of Stewart and Plotkin (2016). Figure 8 seems to confirm this interesting relation insofar as it establishes that the cooperation-induced efficiency increases with decreasing the size of the interacting networks. However, Figure 8 shows that there exists a small size, *M* = 100, at which the efficiency of information transport from one to another self-organizing network is maximal. The heuristic interpretation of this effect is that *temporal complexity* is a finite size property with Δζ proportional to $1/\sqrt{M}$, as shown in Figure 2, namely, with a dependence on the number of units even more significant than in the case of ordinary criticality (Beig et al., 2015), thereby explaining why the communication efficiency increases upon decreasing *M*.

Temporal complexity is the signature of criticality that we adopt, rather than avalanche size, to reveal criticality in the case of self-organization as well as in the case of criticality generated by the fine tuning of the control parameter *K*. In the case of this paper as we have shown earlier with the help of Figures 1, 3, the fluctuating field may be *K* itself, which, as we have seen in the case of individual choice fluctuates around *K* ≈ 1.8, when *M* = 100. Figure 5 shows that the region with μ = 1.3 decreases with increasing *M* and that at *M* = 25 a Poisson shoulder emerges, implying that temporal complexity is lost. Therefore, it explains the interesting result that an optimal size exists, at which the efficiency of information transport becomes maximal. In other words, the intensity of the complex fluctuations transmitting information increases with decreasing *M*, but an excessively small value of *M* annihilates their temporal complexity.

It is important to stress that *M* = 100 depends also on the parameters defining the Prisoners' Dilemma game. The weakening of cooperation with the increase of the number of players is a subject of interest, see Hauert and Schuster (1997) as well as Stewart and Plotkin (2016), thereby generating the issue of establishing if there exists an optimal size of the number of interacting units (Nosenzo et al., 2015). We cannot rule out that a more refined treatment of the dependence on the parameters of the Prisoner's Dilemma game may lead to an optimal value of *M* much smaller than *M* = 100 of Figure 8. However, the emergence of a waiting time PDF with IPL seems to prevent us from accounting for the results of the experimental investigation of Nosenzo et al. (2015), setting *M* = 2 as the optimal size for cooperation emergence.

## 7. Conclusions

This research work has been stimulated by the manifesto of computational science (Conte et al., 2012) listing *scaling* and *criticality* as two crucial aspects of computational social science. Herein, criticality was not forced upon the networks by setting the suitable value *K* for the imitation strength, as done in earlier work (Turalska et al., 2011; Zare and Grigolini, 2013; Mahmoodi and Grigolini, 2017). The critical value of *K* is spontaneously reached without artificially enhancing altruism, but assuming that each unit selects the value of *K* assigning to themselves the maximal benefit.

It is important to notice that the SOTC condition is reached regardless of whether we adopt the individual or the global choice rules. The global choice rule implies the existence of a leader and consequently of intelligence driving the social system. The fact that criticality is spontaneously generated adopting also the individual choice rule is a compelling indication that the model of this paper can be interpreted also as a spontaneous transition to the condition of *swarm intelligence*.

The connection between criticality and swarm intelligence was widely discussed in Vanni et al. (2011) and Luković et al. (2014). Due to the criticality-induced long-range correlation a small number of lookout birds, perceiving the arrival of a predator and changing flying direction, thanks also to the simultaneous occurrence of crucial events, do succeed in exerting a strong influence on the swarm, enough to make the swarm change direction. This form of collective intelligence, due to the criticality-induced long-range space correlation is the intuitive explanation of the surprising fact that the local interaction between the single individuals and their four nearest neighbors generates the emergence of cooperation at the level of the whole network. This is due to the fact that the SOTC is equivalent to a spontaneous transition to the condition of swarm intelligence.

Notice that *K* in the earlier work of our group was interpreted as a form of blind imitation (West et al., 2014). On the other hand the SOTC leads us to interpret *K*, the intensity of which is decided by the individuals on the basis of their benefit as the origin of intelligence and altruism, rather than a form of blind imitation. This model does not require to go through (Nowak and May, 1992) to prevent the infiltration of defectors in cooperation clusters but it establishes the emergence of cooperation with the mere use of the Prisoner's Dilemma payoff thereby connecting the evolution of cooperation (Axelrod, 2006) with the search of agreement between the individuals and their nearest neighbors.

**Figure 9:**
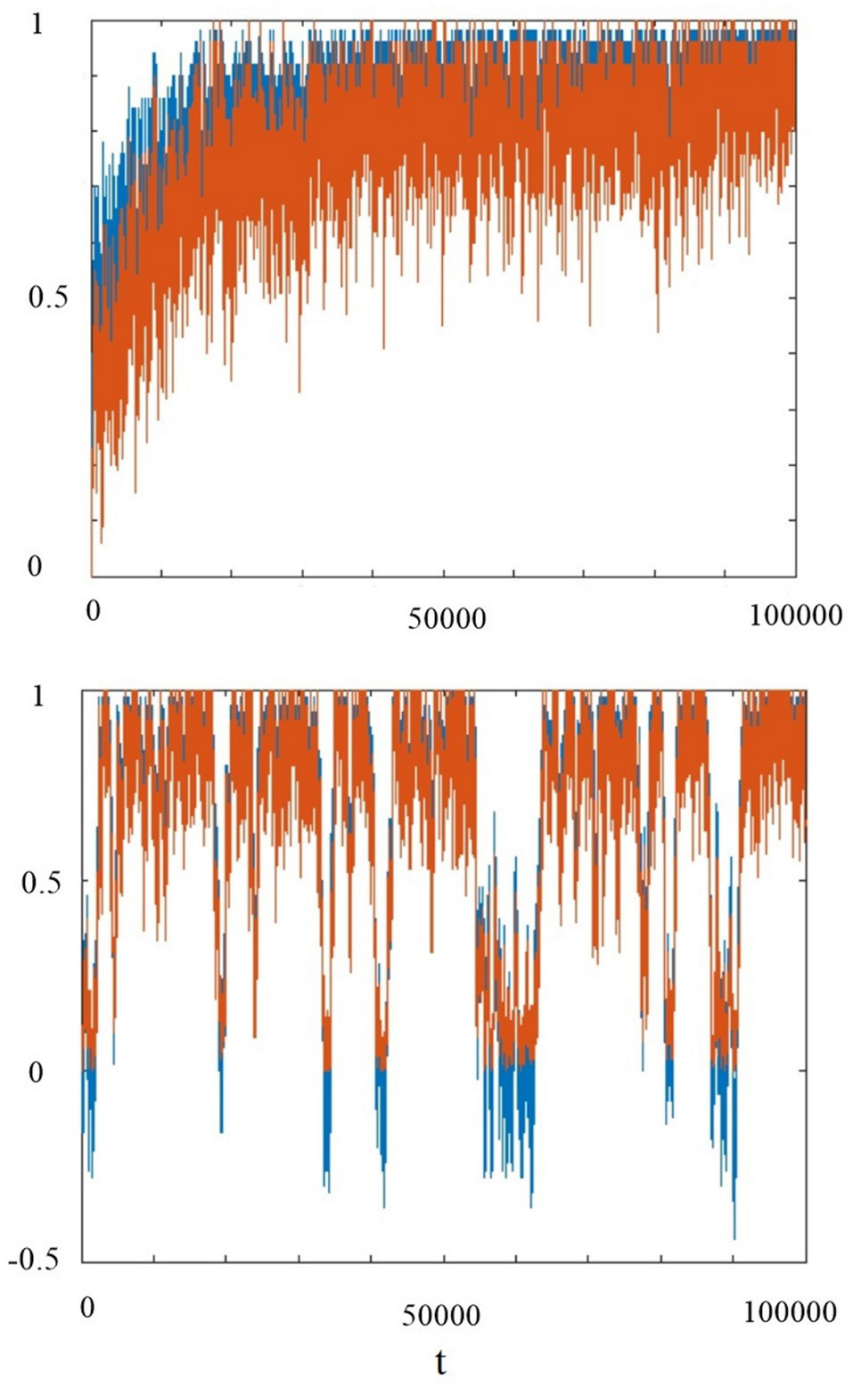
The blue curves denote the mean field *x*(*t*). The red curves denote the ratio of number of cooperator units that are surrounded by 4 cooperators to the total number of units. The top panel refers to the individual choice and the bottom panel to the global choice. For both choices we adopted the parameters: *T* = 1.5, χ = 4, *K*(1) = 0.5, *M* = 100.

The global choice does not prevent the occurrence of organization collapses of the system, as clearly illustrated by Figure 9. This figure indirectly evaluates the size of clusters of cooperators by counting the number of cooperator units surrounded by four cooperators. We see that the global condition is characterized by frequent collapses corresponding to the fragmentation of the clusters of cooperators, whereas the individual self-organization is not affected by these collapses.

As mentioned earlier, the global choice rule is a form of top-down process, implying the action of a benevolent dictator (Helbing and Pournaras, 2015; Helbing, 2017). Thus, as earlier, the SOTC model strongly supports the conjectures of Helbing and Pournaras (2015) and Helbing (2017).

We stress that the SOTC model of this paper can also be interpreted as a contribution to evolutionary game theory explaining the origin of morality. In fact, we conjecture that the swarm intelligence emerging from the SOTC bottom up process may be a form of collective mind (Grigolini et al., 2015) implying that all the individuals have the intuitive feeling that cooperation is the convenient choice (Rand, 2016). Using the terms adopted by (Rand, 2016) we may identify his intuitive decision making with the choice of imitation strength and his deliberative decision making as a direct adoption of the Prisoner's Dilemma game. The theoretical prediction made by Rand that “deliberation will undermine pure cooperation” seems to fit the observation (Mahmoodi and Grigolini, 2017) that the lack of criticality disrupts the Nowak and May network reciprocity (Nowak and May, 1992).

The SOTC model of this paper is highly simplified and ignores, for instance, the cost of the cooperation choice, which is explicitly taken into account, for instance, by Archetti and and Scheuring (2016). We expect that the inclusion of the cost may have an effect equivalent to increasing the incentive to cheat and that this will affect the time scale for the emergence of cooperation. In other words, the transition to the complex fluctuations that in Figure 1 is so fast as to be not visible in the scale of that figure, may become significantly slower, without changing, however, the main properties of temporal complexity and complexity matching, illustrated in this paper. We conjecture that this and other issues, including those of anthropological interest, may be included in the composite network without affecting the main conclusion that this form of SOTC has a general validity, ranging from the random growth of surfaces (Failla et al., 2004) to sociology.

It is also important to stress that SOC is invoked by an increasing number of researchers in the field of complexity but its connection with the open field of phase transitions in systems of small size is not yet properly taken into account. This paper affords a contribution to this still open research subject that hopefully may attract the attention of the researchers in the field of complexity, from biology to anthropology and from neurophysiology to sociology.

## Author contributions

KM: Research and simulation. BW and PG: Advisors.

### Conflict of interest statement

The authors declare that the research was conducted in the absence of any commercial or financial relationships that could be construed as a potential conflict of interest.

## References

[B1] Aquino G. Bologna M. Grigolini P. West B. J. (2010). Beyond the death of linear response: 1/f optimal information transport. Phys. Rev. Lett. 105:040601. 10.1103/PhysRevLett.105.040601 20867831

[B2] Archetti M. Scheuring I. (2016). Evolution of optimal Hill coefficients in nonlinear public goods games. J. Theor. Biol. 406:73. 10.1016/j.jtbi.2016.06.030 27343626

[B3] Axelrod R. (2006). The Evolution of Cooperation. Revised Edn. New York, NY: Basic Books.

[B4] Axelrod R. Hamilton W. D. (1981). The Evolution of Cooperation. Science 211:1390. 10.1126/science.7466396 7466396

[B5] Bak P. Chen K. (1989). The physics of fractals. Phys. D 38:5 10.1016/0167-2789(89)90166-8

[B6] Beig M. T. Svenkeson A. Bologna M. West B. J. Grigolini P. (2015). Critical slowing down in networks generating temporal complexity. Phys. Rev. E. 91:012907. 10.1103/PhysRevE.91.012907 25679682

[B7] Conte R. Gilbert N. Bonelli G. Cioffi-Revilla C. Deffuant G. Kertesz J. (2012). Manifesto of computational social science. Eur. Phys. J. Special Topic. 214:325 10.1140/epjst/e2012-01697-8

[B8] Failla R. Ignaccolo M. Grigolini P. Schwettmann A. (2004). Random growth of interfaces as a subordinated process. Phys. Rev. E. 70:010101. 10.1103/PhysRevE.70.010101 15324032

[B9] Gintis H. (2014). The Bounds of Reason: Game Theory and the Unification of the Behavioral Sciences. Princeton, NJ: Princeton University Press.

[B10] Grigolini P. Piccinini N. Svenkeson A. Pramukkul P. Lambert D. West B. J. (2015) From neural social cooperation to the global emergence of cognition. Front. Bioeng. Biotechnol. 3:78. 10.3389/fbioe.2015.00078 26137455 PMC4468630

[B11] Hauser O. P. Rand D. G. Peysakhovich A. Nowak M. A. (2014). Cooperating with the future. Nature 511, 220–223. 10.1038/nature13530 25008530

[B12] Hauert C. Schuster H. G. (1997). Effects of increasing the number of players and memory size in the iterated Prisoner's Dilemma: a numerical approach. Proc. Biol. Sci. 264:513 10.1098/rspb.1997.0073

[B13] Helbing D. (2017). The Dream of Controlling the World-and Why it Often Fails. Working Paper.

[B14] Helbing D. Pournaras E. (2015). Society: build digital democracy. Nature 527:7576. 10.1038/527033a 26536943

[B15] Hesse J. Gross T. (2014). Self-organized criticality as a fundamental property of neural systems. Front. Sys. Neurosci. 8:166. 10.3389/fnsys.2014.00166 25294989 PMC4171833

[B16] Lipiello E. De Arcangelis L. Godano C. (2005). Memory in self-organized criticality. Europhys. Lett. 72:678 10.1209/epl/i2005-10292-x

[B17] Kim J. M. Bray A. J. Moore M. A. (1992). Domain growth, directed polymers, and self-organized criticality. Phys. Rev. A. 45:8546. 9906953 10.1103/physreva.45.8546

[B18] Lukovic M. Grigolini P. (2008). Power spectra for both interrupted and perennial aging processes. J. Chem. Phys. 129:184102. 10.1063/1.3006051 19045381

[B19] Luković M. Vanni F. Svenkeson A. Grigolini P. (2014). Transmission of information at criticality. Phys. A 416:430 10.1016/j.physa.2014.08.066 21902433

[B20] Mafahim J. U. Lambert D. Zare M. Grigolini P. (2015). Complexity matching in neural networks. New J. Phys. 17:015003 10.1088/1367-2630/17/1/015003

[B21] Mahmoodi K. Grigolini P. (2017). Evolutionary game theory and criticality. J. Phys. A Math. Theor. 50:015101 10.1088/1751-8113/50/1/015101

[B22] Martinello M. Hidalgo J. di Santo S. Maritan A. Plenz D. Muñoz M. A. (2017). Neutral theory and scale-free neural dynamics. arXiv:1703.05079 [q-bio.NC]

[B23] Nosenzo D. Quercia S. Sefton M. (2015). Cooperation in small groups: the effect of group size. Exp. Econ. 18:4 10.1007/s10683-013-9382-8

[B24] Nowak M. A. May R. M. (1992). Evolutionary games and spatial chaos. Nature 359:826 10.1038/359826a0

[B25] Piccinini N. Lambert D. West B. J. Bologna M. Grigolini P. (2016). Non ergodic complexity management. Phys. Rev. E. 93:062301 10.1103/PhysRevE.93.062301 27415274

[B26] Rand D. G. (2016) Cooperation, fast slow: meta-analytic evidence for a theory of social heuristics self-interested deliberation. Psychol. Sci. 1:15 10.1177/0956797616654455. 27422875

[B27] Rosenfeld S. (2013). Global consensus Theorem and self-Organized criticality: Unifying principles for Understanding self-Organization, swarm Intelligence and Mechanisms of carcinogenesis. Gene Regul. Sys. Biol. 7:23. 10.4137/GRSB.S10885 23471309 PMC3583443

[B28] Stewart A. Plotkin J. B. (2016). Small groups and long memories promote cooperation. Sci. Report 6:26889. 10.1038/srep26889 27247059 PMC4887980

[B29] Turalska M. West B. J. Grigolini P. (2011). Temporal complexity of the order parameter at the phase transition. Phys. Rev. E. 83:061142. 10.1038/srep01371 21797337

[B30] Vanni F. Luković M. Grigolini P. (2011). Criticality and Transmission of Information in a Swarm of Cooperative Units. Phys. Rev. Lett. 107:078103. 10.1103/PhysRevLett.107.078103 21902433

[B31] Vilone D. Ramasco J. J. Sánchez A. San Miguel M. (2012). Social and strategic imitation: the way to consensus. Sci. Rep. 2:686. 10.1038/srep00686 23008751 PMC3449285

[B32] Vilone D. Ramasco J. J. Sánchez A. San Miguel M. (2014). Social imitation versus strategic choice, or consensus versus cooperation, in the networked Prisoner's Dilemma. Phys. Rev. E. 90:022810. 10.1103/PhysRevE.90.022810 25215784

[B33] Watkins N. W. Pruessner G. Chapman S. C. Crosby N. B. Jensen H. J. (2016). 25 Years of self-organized criticality: concepts and controversies. Space Sci. Rev. 198, 3–44. 10.1007/s11214-015-0155-x

[B34] West B. J. Turalska M. Grigolini P. (2014). Networks of Echoes: Imitation, Innovation and Invisible Leaders. New York, NY: Springer International.

[B35] Zare M. Grigolini P. (2013). Citicality and avalanches in neural networks. Chaos Solitons Fractals 55:80 10.1016/j.chaos.2013.05.009

[B36] Zapperi S. Lauritsen K. B. Stanley H. E. (1995). Self-Organized branching processes: mean-field theory for avalanches. Phys. Rev. Lett. 75:4071. 10.1103/PhysRevLett.75.4071 10059807

